# Evaluation of the efficacy of the automatic ozone decontamination device in a real-life setting of an intravitreal injection room

**DOI:** 10.1016/j.infpip.2026.100507

**Published:** 2026-01-08

**Authors:** D. Salom, A. Adloff-Alonso, M. Salvador Aguilá, C. Martínez-Rubio, A. Janicka-Caulineau, M. Moro-Muniz, R. Rodrigo

**Affiliations:** a Department of Ophthalmology, Manises Hospital, Manises, Spain; b Department of Preventive Medicine and Public Health, Manises Hospital, Manises, Spain; c Catholic University of Valencia (UCV), Faculty of Medicine and Health Sciences, Valencia, Spain; d Group of Pathophysiology and Therapies for Vision Disorders, Príncipe Felipe Research Center (CIPF), Valencia, Spain; e Biomedical Research Networking Center in Rare Diseases (CIBER-ER), Institute of Health Carlos III, Valencia, Spain

**Keywords:** Intravitreal injection, Ozone, Automatic room disinfection

## Abstract

**Objective:**

The objective of this study was to evaluate the efficacy of an ozonation system for disinfecting intravitreal injection rooms.

**Design:**

A prospective intervention pilot study was conducted with a ‘before-and-after’ design.

**Setting:**

The study was conducted in an intravitreal injection room at Hospital de Manises.

**Methods:**

Air and surface samples were taken before and after ozonation using a remote-controlled ozonation system. The room was treated with ozone at a concentration of 9–10 ppm for 20 min. Measurements were taken immediately after, 12 h, and 24 h post ozonation. Statistical analysis was performed using the Wilcoxon signed-rank test.

**Results:**

At 24 h post ozonation, significant reductions in microbial load were observed: in the centre of the room, the bacterial load reduced from >250 colony-forming units (cfu) to 10 cfu (96%) and fungi from 14 cfu to <1 cfu (93.1%) (*P* < 0.05); in the diffuser, the bacterial load reduced from 61 cfu to 24 cfu (60.7%) and fungi from 6 cfu to <1 cfu (100%) (*P* < 0.05) and on the procedure chair, the bacterial load reduced from 51 cfu to 7 cfu (86.3%) and fungi from 6 cfu to 3 cfu (50%) (*P* < 0.05).

**Conclusions:**

In a dedicated intravitreal injection room, a remotely controlled ozonation cycle at 9–10 ppm for 20 min was associated with substantial short-term reductions in airborne and selected surface microbial load under unoccupied conditions. Automated ozonation may complement routine cleaning and ventilation to maintain low environmental bioburden; its clinical impact remains to be determined.

## Introduction

Intravitreal injections have become a crucial intervention in modern ophthalmology, primarily used to administer medications directly into the vitreous of the eye. This procedure is particularly relevant for treating various ocular conditions such as age-related macular degeneration (AMD), diabetic macular oedema and central retinal vein occlusion. Anti vascular endothelial growth factor (Anti-VEGF) medication injections have revolutionized the treatment of AMD, significantly improving patients’ visual outcomes [1]. Similarly, in diabetic retinopathy, these injections have proven to be highly effective in controlling diabetic macular oedema and preventing disease progression [2]. Central retinal vein occlusion, another severe condition that can lead to sudden vision loss, is also efficiently managed with intravitreal injections, improving visual outcomes and quality of life for affected patients [3]. Due to the chronic nature of these increasingly prevalent pathologies and the need for repeated treatments over time, there has been an exponential increase in the number of intravitreal injections performed in our hospitals, necessitating the creation of specific spaces and care circuits for these patients.

Despite significant benefits, intravitreal injections are not without risks. One of the most serious risks is endophthalmitis, an intra-ocular infection that can result in permanent vision loss if not treated promptly and adequately [4]. Endophthalmitis can be caused by the introduction of bacteria into the eye during the procedure, highlighting the importance of performing this procedure in a sterile and controlled environment. The incidence of postinjection endophthalmitis varies between 0.01% and 0.05%, depending on the environment and aseptic techniques used [5]. Although these rates are relatively low, the impact of an infection is considerable, both in terms of morbidity and the costs associated with treating the complications. Therefore, optimizing aseptic conditions in the rooms where these intravitreal injections are performed is crucial to minimizing these risks.

Hospital environments are complex and host a variety of pathogens that can survive on inanimate surfaces for extended periods. These pathogens can be transmitted through contact with contaminated surfaces, potentially leading to nosocomial infections [6]. Nosocomial infections are a significant concern in health care, affecting approximately 6.5% of hospitalized patients and potentially causing severe complications and prolonged hospital stays [7].

Adequate surface disinfection in hospital environments is challenging due to several factors. First, the effectiveness of traditional disinfection methods largely depends on correct and consistent implementation by cleaning staff [8]. Proper selection of disinfectants, thorough application and adherence to exposure time are crucial to ensuring effective pathogen elimination. However, studies have shown that routine cleaning practices often fail to achieve adequate disinfection, leaving critical surfaces contaminated [9].

Ozone (O_3_) has been identified as a potentially effective disinfectant due to its oxidizing properties. Ozone can destroy the cell membranes of bacteria, moulds and yeasts by breaking the phospholipid chains that compose them, causing the death of these micro-organisms [10]. A significant advantage of using ozone is that the only by-product of its decomposition is oxygen (O_2_), making it an environmentally safe and efficient method. However, the use of ozone in hospital disinfection must be carefully controlled to avoid health risks.

The remote-controlled ozonation system (RCOS) allows precise control and measurement of ozone concentrations in real time and remotely. This capability ensures that ozone is applied effectively and safely, minimizing risks to patients and healthcare personnel. This study aims to evaluate the efficacy of this technology in disinfecting an intravitreal injection room under real-life conditions following an injection session in 36 patients.

## Methods

This is a pilot prospective intervention study designed to evaluate the efficacy of the RCOS developed by Hi-Tech Ozone® (Valencia, Spain) in an intravitreal injection room. The study follows a ‘before-and-after’ design, comparing environmental and surface contamination conditions before and after the application of ozone.

The study was conducted in the intravitreal injection room at Hospital de Manises. This room is specifically designed for performing intravitreal procedures, ensuring a controlled and suitable environment for their execution. The intravitreal injection room has no windows and is a dedicated ∼10-m^2^ procedural room with a mechanical air ventilation diffuser. The room is located in the presurgical area of the ambulatory surgery unit. It is a positive-pressure room, and the ventilation system is equipped with High-Efficiency Particulate Air (HEPA) filtration. Between the end of the clinical list and each postozonation sampling time, the room remained unoccupied and access was restricted. To minimize operator-induced contamination during sampling, air and surface collections were carried out with the personal protective equipment on (surgical mask and sterile gloves) following hand hygiene. Surface samples were obtained by a single trained investigator. Air and surface outcomes are analysed and reported separately. There was no exposure of patients or healthcare personnel to ozone during the disinfection process, ensuring their safety at all times.

Air and surface samples were collected to determine the count of aerobic micro-organisms, moulds and yeasts, as well as the identification of fungi. For environmental samples, air samples were taken using a surface air system (SAS) at two different points in the room. One thousand liters of air was analysed on sterile replicate organism detection and counting (RODAC) plates with tryptic soy agar (TSA); (Scharlab, Barcelona, Spain) for bacteria and Rose Bengal agar for fungi at a suction flow rate of 60 L per minute in two locations, in the centre of the room and near the air ventilation diffuser.

For surface samples, samples were taken from the procedure chair using contact plates with TSA agar for bacteria and Bengal Rose agar for fungi. Surface samples were collected under the same temporal conditions as environmental samples, allowing a direct comparison between the microbial load in the air and critical surfaces in the room.

Four measurements were taken: the first right after the intravitreal injection session, the second immediately after ozonation, as well as 12 and 24 h after ozonation.

The RCOS was used for ozonation. The room was closed and sealed to reach an ozone concentration of 9–10 ppm for at least 20 min, and the room was re-opened when safe ozone concentration levels below 0.04 ppm were reached. Notably, thanks to the unique capability of the RCOS to remotely control the exact ozone levels in the environment, we were able to ensure that when opening the intravitreal injection room, it was completely safe. Additionally, an ozone capture and destruction system was installed to accelerate ozone elimination as much as possible, thereby ensuring the safety of the facilities, healthcare personnel and patients at all times.

The collected data were analysed using appropriate statistical methods. The Wilcoxon signed-rank test was used for paired samples to compare micro-organism counts before and after the intervention. The statistical software STATPLUS (AnalystSoft Inc. Virginia, USA) was used for all analyses.

We acknowledge that an additional sampling day without ozone exposure would be required to quantify ozone-specific effects. This pilot study, with a pragmatic before-and-after design, does not allow the independent contributions of ventilation, room occupancy or ozone to be distinguished. Accordingly, our aim in this study was to characterize the potential contribution of ozonization when added to the existing decontamination workflow, which also includes room vacancy and continuous mechanical ventilation.

## Results

The initial results showed a high concentration of bacteria and fungi in all locations: in the centre of the room, >250 colony-forming unit (cfu) of bacteria and 14 cfu of fungi (*Penicillium* and *Ulocladium* spp.); in the diffuser, 61 cfu of bacteria and 6 cfu of fungi (*Penicillium* and *Monilia* spp.) and on the procedure chair, 51 cfu of bacteria and 6 cfu of fungi (yeasts, sterile mycelium and *Penicillium* spp.).

**Figure 1:**
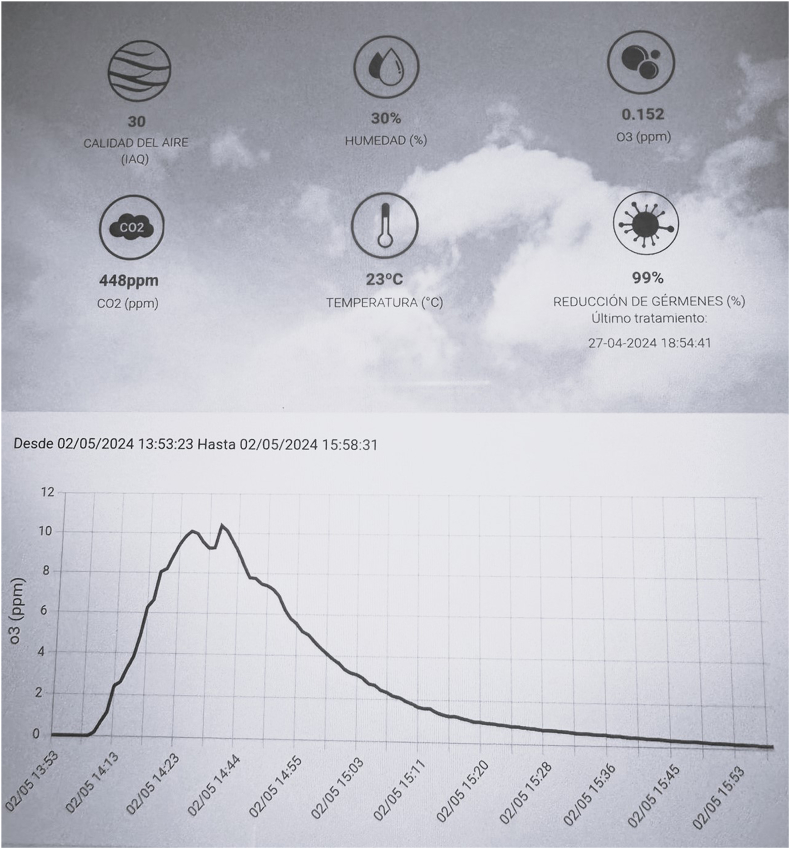
**Monitoring of environmental conditions and ozone concentration during the ozonation process in the intravitreal injection room, using the remote-controlled ozonation system for real-time and remote representation**. The graph shows the increase and decrease of ozone (O_3_) levels in ppm from 13:53:23 to 15:58:31 on 02/05/2024. Recorded environmental parameters include air quality (IAQ) at 30, humidity at 30%, CO_2_ at 448 ppm, temperature at 23 °C and a 99% reduction in germs. The maximum ozone concentration reached approximately 10 ppm and was maintained during the treatment before decreasing to safe levels.

Treatment levels of 9–10 ppm were reached within 20 min from the start of ozone production and maintained for at least 20 min. Ozone concentration reduced to safe levels of 0.04 ppm approximately 3.5 h after treatment began (Figure 1).

Immediately after ozonation, the results showed a drastic reduction in microbial load in the centre of the room, the diffuser and the procedure chair: in the centre of the room, the bacterial load reduced to 22 cfu (91.2%) and fungi to 11 cfu (21.4%) (*Ulocladium* spp.) (*P* < 0.05); in the diffuser, the bacterial load reduced to 13 cfu (78.7%) and fungi to 9 cfu (−50%) (*Ulocladium* and *Penicillium* spp.) (*P* < 0.05) and on the procedure chair, the bacterial load reduced to 23 cfu (54.9%) and fungi to 2 cfu (66.7%) (*Penicillium* spp.) (*P* < 0.05).

At 12 h post ozonation, there was a continued decrease in microbial load: in the centre of the room, the bacterial load reduced to 18 cfu (92.8%) and fungi to <1 cfu (93%) (*P* < 0.05); in the diffuser, bacteria remained at 13 cfu (78.7%) and the fungal load reduced to 3 cfu (50%) (*Ulocladium* spp.) (*P* < 0.05) and on the procedure chair, the bacterial load reduced to 16 cfu (68.6%) and fungi to <1 cfu (83.3%) (*P* < 0.05).

At 24 h, there was a continued reduction in microbial load: in the centre of the room, the bacterial load reduced to 10 cfu (96%) and fungi remained at <1 cfu (93.1%) (*P* < 0.05); in the diffuser, bacteria slightly increased to 24 cfu (60.7%), but fungi remained at <1 cfu (100%) (*P* < 0.05) and on the procedure chair, the bacterial load reduced to 7 cfu (86.3%) and fungi remained at 3 cfu (50%) (*Penicillium* spp.) (*P* < 0.05). Table I summarizes all the results.

**Table I:** Summary of microbial load results after ozonation with remote-controlled ozonation system

Location	Sampling method	Initial (cfu)	Immediately after (cfu, %)	12 h (cfu, %)	24 h (cfu, %)
Centre of the room	Air sampling	Bacteria: >250 Fungi: 14	Bacteria: 22 (91.2%) Fungi: 11 (21.4%)	Bacteria: 18 (92.8%) Fungi: <1 (93%)	Bacteria: 10 (96%) Fungi: <1 (93.1%)
Diffuser	Air sampling	Bacteria: 61 Fungi: 6	Bacteria: 13 (78.7%) Fungi: 9 (−50%)	Bacteria: 13 (78.7%) Fungi: 3 (50%)	Bacteria: 24 (60.7%) Fungi: <1 (100%)
Procedure chair	Surface sampling	Bacteria: 51 Fungi: 6	Bacteria: 23 (54.9%) Fungi: 2 (66.7%)	Bacteria: 16 (68.6%) Fungi: <1 (83.3%)	Bacteria: 7 (86.3%) Fungi: 3 (50%)

cfu: colony-forming units; %: percentage reduction from the initial value.

## Discussion

The analysis of the results reveals a significant reduction in microbial load in the intravitreal injection room following ozone intervention. Immediately after ozonation, there was a reduction of 91.2% in bacteria and 21.4% in fungi in the centre of the room. This trend continued, with reductions of 96% in bacteria and 93.1% in fungi at 24 h, consistent with the initial bioburden decrease achieved after ozonation and the subsequent effects of room vacancy and continuous mechanical ventilation.

The reduction in the diffuser showed a decrease of 78.7% in bacteria and an initial increase of 50% in fungi immediately after ozonation. At 24 h, the bacterial load reduced by 60.7% and fungi by 100%. This variability could be attributed to environmental factors and ozone distribution in the room, warranting further investigation.

Surface cultures on the procedure chair also showed a significant reduction in microbial load. Immediately after ozonation, there was a reduction of 54.9% in bacteria and 66.7% in fungi. At 24 h, the reductions were 86.3% in bacteria and 50% in fungi. These suggest that ozone could be effective not only for air disinfection but also for critical surfaces in the injection room.

However, it is noteworthy that the micro-organisms most frequently implicated in endophthalmitis, such as *Staphylococcus epidermidis* and *Staphylococcus aureus*, were not characterized in the cultures either before or after ozonation, and these are the most frequent causes of endophthalmitis [11]. It would be interesting to evaluate the efficacy of ozone against these specific pathogens in future investigations.

As ozone naturally decomposes within a short time after the treatment cycle, we cannot determine whether the reductions observed in cfu counts reflect true microbicidal activity—i.e., irreversible killing of micro-organisms—or whether they represent a transient microbistatic effect, in which surviving organisms may have been temporarily inhibited and therefore unable to grow at the time of sampling. Distinguishing between these mechanisms would require targeted viability assays, which were beyond the scope of the present environmental evaluation.

This study evaluated environmental bioburden (air and selected surface cfus) rather than clinical outcomes such as endophthalmitis. We therefore do not infer a reduction in infection risk from these data. Prevention of postinjection endophthalmitis is primarily driven by strict aseptic technique and maintenance of a sterile field; environmental decontamination should be considered complementary. Regarding airborne contribution, the exposed area of the injection needle and the brief pre-injection exposure likely limit the role of airborne microbes. Nonetheless, we observed marked reductions in airborne and surface cfus immediately after ozonation and at 12–24 h under unoccupied conditions. Whether environmental improvements translate into fewer clinical infections requires adequately powered studies.

Compared with other disinfection measures, such as vaporized hydrogen peroxide and ultraviolet radiation, ozone presents advantages in terms of effectiveness, safety and environmental friendliness. Previous studies have demonstrated the effectiveness of ozone in disinfecting surfaces and air in hospital environments. For example, Davies *et al.* [10] found that ozone disinfection was effective against *Clostridium difficile* in the hospital environment. Likewise, Knobling *et al.* [12] evaluated two automated disinfection devices under real conditions and found a significant reduction in microbial load. These studies support the findings of the present study, underscoring the effectiveness of ozone as a disinfectant in clinical settings. The present investigation is pioneering in evaluating the use of ozone specifically in an intravitreal injection room, providing valuable data that can be applied to other similar hospital environments, especially because it was conducted under real-life conditions.

Implementing automated ozone disinfection systems could significantly improve hygiene in intravitreal injection rooms and other critical hospital environments. This, in turn, could reduce the risk of nosocomial infections and improve patient safety. Additionally, the RCOS, which allows precise control and monitoring of ozone concentrations, offers a safe and efficient solution for hospital disinfection. This approach is not only effective but also environmentally friendly as ozone decomposes into oxygen, leaving no harmful chemical residues.

This study underscores the importance of implementing advanced technologies, such as ozone disinfection, in the fight against nosocomial infections. Adopting these technologies can play a crucial role in improving patient safety and the quality of healthcare services. The integration of ozone systems into hospital cleaning routines should be considered as part of a comprehensive infection control strategy. This includes coordination with other disinfection measures and optimization of existing cleaning protocols.

Although the results are promising, this study presents some limitations that should be considered. First, the variability observed in the reduction of contaminants in the diffuser suggests that ozone distribution may not be uniform throughout the room. This could be due to factors such as ventilation and air flow, which were not exhaustively controlled in this study. Future research should consider these factors to optimize ozone efficacy. Additionally, it may be necessary to increase the exposure time to be more effective since 20 min may be insufficient. Moreover, sampling relied on a standardized SAS air sampler (1000 L at 60 L/min) at two fixed locations (room centre and near the ventilation diffuser) and contact plates on the procedure chair at four time points (post session, immediately post ozonation, 12 h and 24 h). We acknowledge the limited spatial replication and single-day sampling. The reductions observed at 12 and 24 h may reflect the combined effect of room vacancy and continuous mechanical ventilation rather than any residual antimicrobial effect of ozone. Future studies incorporating multi-day sampling and comparison between days with and without ozone exposure will be essential to determine the specific contribution of ozone within the workflow. Finally, the study was conducted in a single intravitreal injection room in one hospital, limiting the generalizability of the results. It would be useful to replicate this study in multiple settings and different hospitals to validate the findings and ensure their generalizability.

In conclusion, this study describes substantial reductions in airborne and surface microbial burden following the decontamination with ozone used in our intravitreal injection room in real-life conditions. These findings indicate that automated ozonation, when integrated into an existing cleaning and ventilation protocol, may serve as a complementary environmental decontamination measure. While ozone decomposes without leaving chemical residues and offers practical advantages for environmental hygiene, the clinical relevance of these environmental changes cannot be inferred from this study and warrants evaluation in future controlled studies.

We recommend some lines of research that this study opens for future investigations, such as conducting multi-centre studies in different clinical settings to validate the results and ensure their generalizability, investigating factors that affect ozone distribution in hospital environments and developing strategies to ensure uniform application, evaluating the long-term impact of ozone use on patient health and the incidence of nosocomial infections and finally comparing the efficacy and safety of ozone with other disinfection methods.

## CRediT authorship contribution statement

**D. Salom:** Writing – review & editing, Writing – original draft, Visualization, Supervision, Resources, Project administration, Methodology, Investigation, Formal analysis, Data curation, Conceptualization. **A. Adloff-Alonso:** Investigation. **M. Salvador Aguilá:** Validation, Project administration, Methodology, Conceptualization. **C. Martínez-Rubio:** Validation, Supervision, Project administration, Methodology, Conceptualization. **A. Janicka-Caulineau:** Investigation. **M. Moro-Muniz:** Investigation. **R. Rodrigo:** Writing – review & editing, Validation, Supervision, Conceptualization.

## Ethics statement

Ethical approval was not required for this study.

## Declaration of generative AI and AI-assisted technologies in the writing process

During the preparation of this work, the authors used ChatGPT 4.0 from Open AI, in order to translate the text from Spanish to English. After using this tool, the authors reviewed and edited the content as needed and take full responsibility for the content of the publication.

## Funding sources

This work was supported by Hi-Tech Ozone (Valencia, Spain).

## Conflict of interest statement

The authors declare no conflict of interest with any commercial product. The authors declare that they have no known competing financial interests or personal relationships that could have appeared to influence the work reported in this paper.
